# Young People Talk About Digital Support for Mental Health: An Online Survey of 15–30‐Year Olds in New Zealand

**DOI:** 10.1111/hex.70001

**Published:** 2024-08-26

**Authors:** Susan M. Garrett, Jo Hilder, Rachel Tester, Abby Dunlop, Tracey Gardiner, Tony Dowell, Soraya Kamau Brady, Nicole Gilbert, Maggie Shippam, Shay Tanirau, Neo Kenny, Caitlin McBride, Joana Wilson, Ellie Rukuwai, Niusha Aryan, Maria Stubbe

**Affiliations:** ^1^ Department of Primary Health Care and General Practice University of Otago Wellington New Zealand; ^2^ Te Paepae Arahi Trust, Lower Hutt Wellington New Zealand; ^3^ Youth Co‐Researcher (CORE) Group University of Otago Wellington New Zealand

**Keywords:** adolescents, digital support, mental health, survey, young people

## Abstract

**Background:**

Mental distress is on the rise for young people, and there are high levels of unmet need for support. Increasingly, young people are engaging with online mechanisms of support to avoid cost and wait times; however, online support does have its limitations. We surveyed young people, 15–30 years of age, in Aotearoa New Zealand to explore their views of digital support for mental health. The aim of this study was to find out from young people what they thought about various types of online support and perceived benefits and drawbacks.

**Methods:**

A cross‐sectional online survey promoted through social media advertising was used. Participants included anyone aged 15–30 years living in Aotearoa New Zealand. The survey ran for 10 weeks between February and May 2022. It included demographic questions and asked about (i) use of digital support for mental health; (ii) what digital support is best used for; (iii) best ways of publicising mental health supports to young people; and (iv) where they would choose to get information about mental health support. Questions were a mix of forced choice and free text. Participants could opt to take part in a follow‐up interview.

**Results:**

Surveys were completed by 1471 participants; two respondents participated in an interview. A total of 641 participants had used digital support before (44%). The most used forms of digital support were websites (*n* = 324) and watching videos (*n* = 260), although these were not necessarily rated as the most helpful. Alternatives that people most wanted to try were podcasts and phone or video consultations with a counsellor or therapist. Drawbacks of digital support included privacy concerns, technical issues, lack of quality and motivation requirements. Benefits included ease of access, anonymity and a non‐threatening starting point.

**Conclusions:**

Digital support has a place in mental health care, but strong sentiment was expressed in favour of real‐life support. It may also be worth investing in more innovative types of digital support such as online performing arts and podcasts.

**Patient or Public Contribution:**

A group of young people were recruited as co‐researchers, had input into survey design, data analysis and interpretation and are co‐authors (list of co‐authors). Survey respondents also included young people with lived experience who are members of the general public.

## 1 Introduction

Rates of mental distress among young people have increased rapidly in recent years, both globally [1] and in Aotearoa New Zealand [2]. The New Zealand Health Survey for 2021/2022 showed that almost one in four (23.6%) young people aged 15–24 years experienced high or very high levels of psychological distress, compared to 5.1% in 2011/2012. Young adults also reported the highest percent of unmet need for professional help in this survey (16.2% for individuals 15–24 years of age and 15.6% for individuals 25–34 years of age) [3].

Technologies for virtual provision of mental health support such as computerised therapies, apps, websites and telehealth (i.e., delivering health services when patients and health professionals are not in the same physical location) are increasingly offered as part of the solution to reducing the burden of mental distress in a cost‐effective and accessible way, especially for young people [4]. Digital tools are seen as having tremendous potential, with claimed benefits of being non‐judgemental and stigma‐free [5], private, flexible, accessible and having far greater uptake than traditional in‐person forms of treatment [6].

Research to date has shown digital tools to be acceptable and effective treatments for the common mental health problems of anxiety and depression in youth [7, 8] as well as for other conditions such as ADHD [9]. Total download numbers for some mental health apps are as high as 42 million [10]. Within New Zealand, Fleming et al estimate that more than 10% of the youth population is likely to have accessed major websites or apps for depression in the last year [6] and some New Zealand research has focused specifically on digital tools for Māori (Indigenous population) and Pasifika youth [11, 12]. Mental Health interventions in the form of well‐designed games can be effective and helpful in improving the mood and hope of participants and teaching participants Cognitive‐Based Therapy (CBT) skills [13]. Benefits have also been reported for mental health service navigation websites, including cost‐effectiveness and resource efficiency [14]. Nevertheless, some questions and issues remain.

It is often assumed that young people will be receptive and enthusiastic about using virtual (i.e., online and telehealth) modes of support, but this assumption needs to be tested. Findings are mixed as to how much young people use different digital resources for mental health. An Irish study found use of social media and mental health apps to be common for young people (18–25 years), but use of formal online resources and online professional counselling less so [15]. The study also found that Google search, recommendations from peers and prior knowledge of services play a role in how resources are located. In contrast, earlier Canadian research [16] found that young people were likely to use information‐based mental health websites when going through a difficult time, but unlikely to use social media for information or help‐seeking due to concern over credibility of information.

Positive results have been found for the use of mental health service navigation websites for young people (18–25 years), including cost‐effectiveness and resource efficiency [14]. Reported barriers to using technology for mental health support by young people include privacy concerns, lack of internet connectivity or technology, along with a preference for in‐person support [17, 18]. Young Australian men were more likely to use technology versus seeking help from professionals, but online interventions needed to be action‐oriented and to incorporate peer influence as opposed to being knowledge‐focussed [19].

Satisfaction in using mental health virtual tools has been reported to increase when created using participatory or co‐design methods for young people (18–25) [14], Indigenous peoples [20] and trans and gender‐diverse young people aged 11–18 years [21]. These groups see themselves represented in co‐designed tools and feel like they have been tailored specifically for them, which translates to acceptance and a willingness to promote to others in their community. Tailoring of digital resources for cultural identity is also important [13, 20]. Evidence is still lacking on how effective online support tools are compared to non‐digital forms of support and/or the degree to which virtual and in‐person modalities might be complementary.

The objectives of this study were to find out from young people:

1. what digital mental health supports they had used and what they thought of them;
2. what supports they would like to try in the future; and
3. what they thought were the best ways of promoting digital mental health supports.

## 2 Methods

The cross‐sectional survey reported here was a component of a larger project exploring youth perspectives on possible tools to help young people navigate virtual mental health support. A youth co‐researcher (CORE) group was established to work on this project, including providing input into the survey. The CORE group comprised a broad mix of youth representatives (aged 18–27) with lived experience of mental distress, and including Māori, Pasifika and Rainbow young people.

### 2.1 Study Population and Recruitment

Young people aged 15–30 years from New Zealand were invited to participate in an anonymous online survey, regardless of whether they had used digital mental health support previously. Following advice from the CORE group, the initial age range of 18–25 years was extended. Recruitment targeted four different demographic groups in three staggered waves utilising social media advertising, with sponsored posts presented to Facebook and Instagram account holders. The three waves targeted specific groups, using adjustments to wording and imagery to appeal to young people from the ethnic and gender groups that were under‐represented in previous waves: Wave 1: All genders aged 15–30 years (14 days); Wave 2: Māori and Pasifika all genders aged 15–30 years and Men aged 18–25 years (15 days); and Wave 3: Pasifika all genders (14 days). This method of targeting is similar to that used in previous studies with social media recruitment [22].

Recruitment took place over 10 weeks between February and May 2022. The recruitment social media posts offered entry into a prize draw for six NZ$50 (USD35) gift cards. On completion of the survey, participants were invited to email the research team to enter the draw, participate in an interview or to receive the results of the survey. The invitation to participate in an interview was to provide an opportunity for further elaboration of responses. Two respondents participated in an online interview, and the transcripts were included with free‐text survey responses as part of a qualitative data set for analysis.

### 2.2 Survey Development and Delivery

The survey questions and recruitment medium drew on past local studies [23, 24, 25] and many questions were based on findings of a local youth mental health evaluation [26]. The CORE group reviewed question content and wording and also gave input into the advertising wording and graphics.

Survey questions covered demographic information (age, gender, ethnicity, location, working, student) and asked about (i) use of digital support for mental health; (ii) what digital support is best used for; (iii) best ways of publicising mental health supports to young people; and iv) where you would choose to get information about mental health support. The survey was a mix of quantitative questions: multiple choice (with single or multiple answers allowed), matrix and rank order questions (rank items in order of preference) and qualitative questions, either space for additional information at the end of a multiple choice (seven questions) or as completely open‐ended responses to elicit ideas without pre‐determining responses (three questions).

‘Digital support’ was defined broadly in the first question as *any kind of mental health support where you are not with a real person in the same space. This can include talking to a real person but over a device (phone, video, text, email, messaging, community forum) as well as anything online such as websites, apps, games or chat‐bots*.

The survey was administered via the Qualtrics online survey platform (Qualtrics, Provo, UT). People clicking on the advertisement link were directed to the information sheet that outlined the purpose of the research, the ways in which the data would be used and gave reassurance about the anonymised, confidential nature of the survey. The statement ‘Clicking on the survey link means you are consenting to participate’ was provided, together with advice that the survey could be ended at any time. A copy of the survey is available in supplementary files.

### 2.3 Data Cleaning and Analysis

The survey data were exported into Microsoft Excel for cleaning, collation and analysis. A range of measures were undertaken to ensure the responses included were legitimate: age and region of residence were checked as the two inclusion criteria, we checked for duplicate IP addresses (which are unique to a computer or device), survey completion times were reviewed and the Qualtrics survey software ‘BOT check’ was performed. Emails requesting prize draw entry were reviewed and no two emails were sent from the same address.

Before analysis, ethnicity data were re‐coded following standardised New Zealand ethnicity protocols to enable reporting on prioritised ethnicity for anyone who reported more than one ethnic group; ethnic groups are prioritised as follows: Māori, Pasifika, Asian, New Zealand European and Other [27]. Descriptive statistics were used to report response frequencies and percentages for reported survey items.

A qualitative thematic analysis [28] was conducted on the free‐text responses and interview transcripts. As a team, we read the body of comments to get a sense of the content and to develop a deductive coding framework. Some comments provided contextual detail to support responses to forced‐choice questions, but much of the free text offered new insights that were not captured elsewhere in the survey. Four members of the team collaboratively coded the comments according to their content (regardless of which question they were associated with in the survey or interview) and identified themes in an iterative inductive process, including discussion to achieve consensus. The qualitative analysis has been integrated into our reporting of the quantitative results.

## 3 Results

The survey advertisement received 3830 unique ‘clicks’, which resulted in 1518 surveys initiated (39.6%, 1518/3830). A total of 977 of these surveys were completed in full, and a further 604 were partially completed. Partially completed surveys were reviewed and a decision was made to include any that answered the first question (following initial demographic questions) ‘Have you ever used digital support for mental health before’. This resulted in inclusion of 1471 participants (with 977 fully complete surveys and 494 partially completed surveys). Responses to three free‐text‐only questions were as follows: Q1 748 respondents (51% of the total sample), Q2 546 respondents (37% of the total sample) and Q3 299 respondents (20% of the total sample). A total of 804 (55%) respondents included at least one free‐text comment.

### 3.1 Participant Characteristics

Table 1 presents the characteristics of the total sample of survey participants. A total of 641 (43.6%) participants reported having used some type of digital mental health support previously. A slightly larger proportion of respondents identified as men (49.7%) than as women (46.4%), with 3.2% identifying as ‘another gender’.

**Table 1:** Characteristics of the survey sample.

	Total sample (*n* = 1471)		Previously used digital support (*n* = 641)	
Characteristics	***n***	**%** a	***n***	**%** b
Age‐band				
15–17 years	542	36.8	238	43.9
18–19 years	294	20.0	102	34.7
20–21 years	194	13.2	77	39.7
22–23 years	199	13.5	84	42.2
24–25 years	158	10.7	66	41.8
26–30 years	115	7.8	74	64.3
Ethnic group				
Māori	213	14.5	91	42.7
Pasifika	81	5.5	32	39.5
Asian	176	12.0	75	42.6
European	952	64.7	418	43.9
Middle Eastern, Latin American, African	31	2.1	17	54.8
Not stated	18	1.2	8	44.4
Gender				
Woman	683	46.4	374	54.8
Man	731	49.7	222	30.4
Another gender^c^	47	3.2	37	78.7
Not stated	10	0.7	8	80.0
Employment/education^d^				
At school or tertiary study	710	48.3	311	43.8
Training/apprenticeship	33	2.2	9	27.3
Working part time	251	17.1	129	51.4
Working full time	208	14.1	103	49.5
Looking for work	94	6.4	59	62.8
Caring for child, someone else	23	1.6	13	56.5
Not working or studying or unable to	33	2.2	18	54.5
Receiving a benefit	27	1.8	19	70.4
Not stated	2	0.1	2	100.0
Where do you live				
In a major urban city	699	47.5	317	45.4
In a regional city	126	8.6	53	42.1
In a small town	90	6.1	46	51.1
In a very small town/rural/remote	60	4.1	22	36.7
Not answered	496	33.7	203	40.9

^a^ Column percentages (denominator = 1471).

^b^ Row percentages.

^c^ Participants could add free text to describe their gender: Agender (2), Demigirl (1), Gender Bender (1), gender‐queer (2), gender‐fluid (4), nonbinary (14) and trans (4).

^d^ Participants could select more than one option, so the total does not sum to 1478.

Individuals 15–19 years of age made up more than half the sample (56.8%). The proportions of respondents identifying as Māori (14.5%) and Pasifika (5.5%) were lower than their population rates of 17.4% and 8%, respectively. Respondents were predominantly from cities but included some from small towns and rural contexts.

### 3.2 Attitudes to Digital Support

#### 3.2.1 Perceived Uses, Benefits and Drawbacks of Digital Support

**Figure 1:**
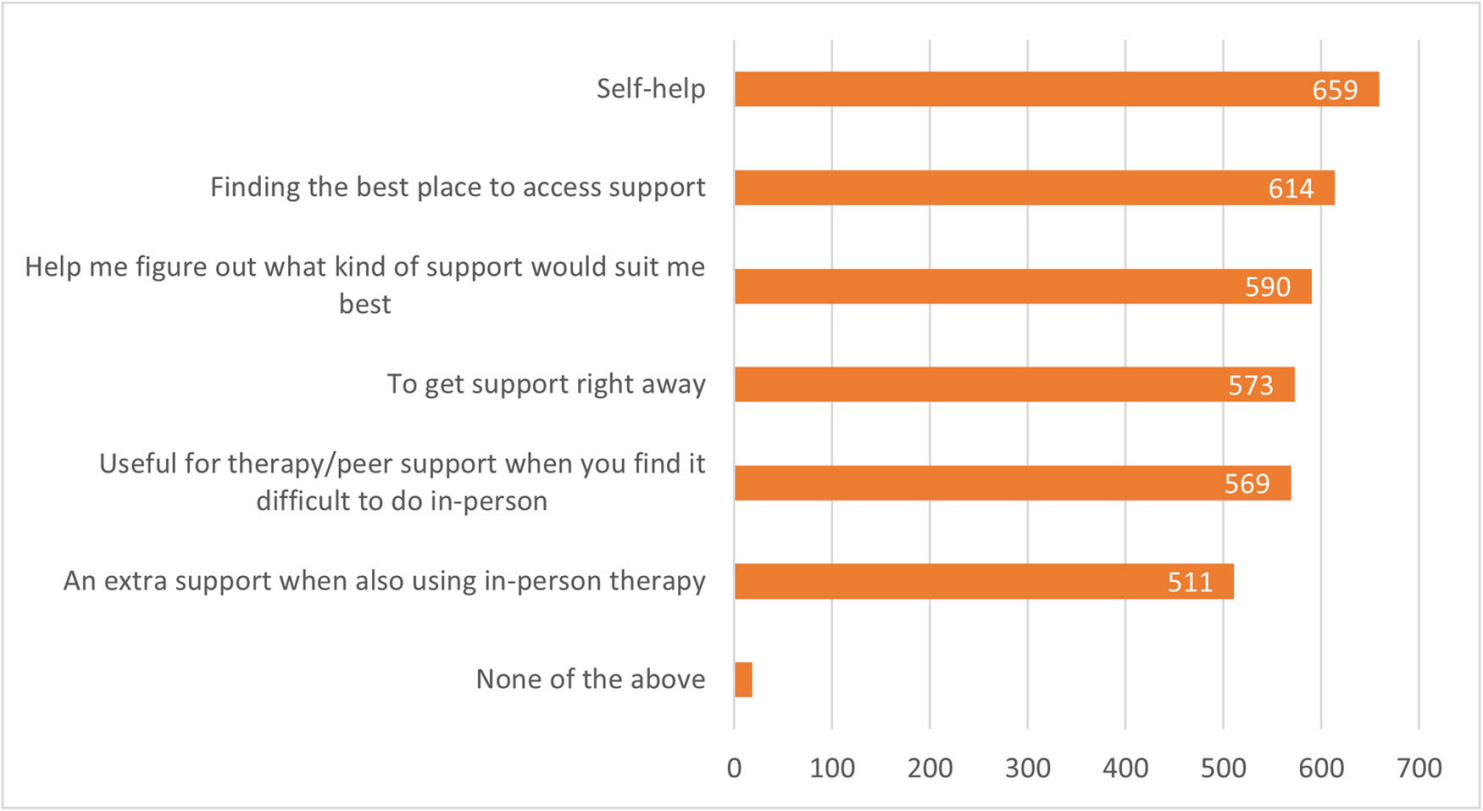
Uses for digital support; participants selected all that apply (*n* = 1111).

Participants were asked to select as many listed options as they thought applied to indicate what they thought digital support might be useful for and could add free‐text comments as an ‘other’ option. Figure 1 shows how many respondents selected each of the options provided.

Free‐text and interview comments provided additional detail on the perceived benefits and drawbacks of digital support. Specific reasons why some people may find it difficult to use in‐person supports (making digital support desirable) included personal reasons such as those who ‘struggle with interacting with people in person’ as well as practical reasons such as COVID‐19 isolation or not being available to attend appointments within working hours and days. Some forms of digital support were also noted as providing welcome anonymity. These benefits (access and anonymity) were identified by those living in rural communities as particularly important, including for avoiding stigma.

Busy time of year, you get run down and stressed and its sort of expected that you could be working up to 12+ days in a row so there isn't that time to get off farm and talk to someone in person so having that ability to utilise an online service would be really great. Particularly with the stigma around particularly young men in rural communities not wanting to seek help. So being able to not be seen going off farm to get help because you're perceived as weak would be really cool too.

(Interview#2: NZE, 28 years, woman)

Some of the more notable comments included the benefit of digital resources and options at an early stage of support‐seeking as a non‐threatening starting point (#161) or elaborated on their utility as a way to figure out what kind of support would suit, while emphasising that the actual support would need to be with ‘a real person’ (#1457):

Sometimes having lower commitment options if you are in the precontemplation or contemplation stages can make it less scary, especially if just scoping out things.

(#161: NZE, 20–21 years, woman)

A good place to start would be a one stop shop that branches people out to the appropriate mental health support that they need ‐ specific to their ‘condition’. A digital form could be the starting place, that eventually leads to connecting with a real person. I don't believe healing should solely be done online, we need human connection.

(#1457: Māori, 26–30 years, woman)

Drawbacks mentioned included issues with trust and privacy when accessing digital support, technical issues, long wait times and poor quality of services.

Digital services were also often negatively compared with in‐person support, and the cost of some digital services was a drawback. Another issue was the need for self‐motivation in order to benefit from them:

I think the main reason most things I've tried on the internet weren't helpful because you need a lot of self‐motivation and dedication, and there isn't anyone to hold you to it apart from yourself.

(#229: European, 15–17 years, woman)

In addition, the ubiquitousness of the digital world, which can be seen as a reason to provide services in this way, can also work against its appeal, as shown in the following brief comment:

Online screen fatigue.

(#743: NZE, 24–25 years, woman)

Finally, an interesting drawback identified by a slightly older participant was the difficulty of observing others doing well online and ‘then trying to understand why I feel “shit”.

Learning to navigate social media as a young person when everyone looks like they're doing really well with life and there's no problems in the background is not easy. Particularly if you're taking all of it as truth because you haven't necessarily been taught that that's [not] the case.

(Interview#2: NZE, 28 years, woman)

#### 3.2.2 Perceptions of Specific Forms of Digital Support

**Figure 2:**
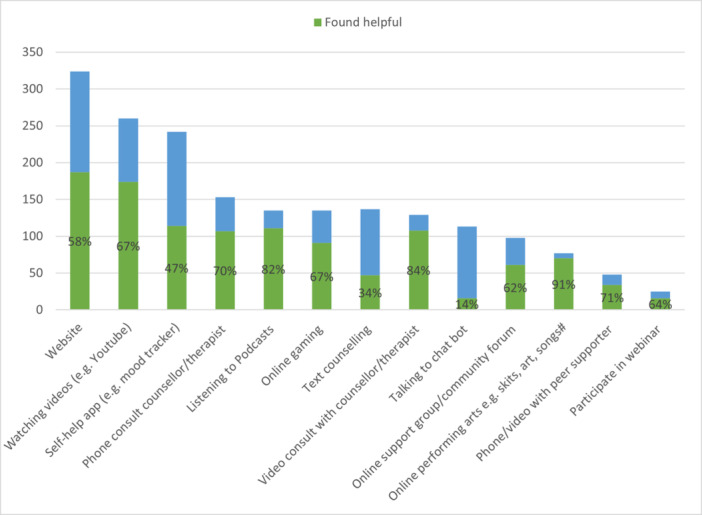
Digital support used (*n*) and proportion (%) who found it to be helpful (*n* = 543).

Those who had used digital support were asked which forms of support they had used and whether they found them helpful. Figure 2 shows the supports in order of how many respondents reported using them. *Browsing a website* and *watching a video* were the most used, and webinars were the least used. The stacked proportions show what proportion of respondents who had used the various supports found them helpful, indicating that some of the lesser used mediums were nevertheless reported as the most helpful for the smaller numbers of those who had used them (*Online performing arts*—91% found helpful and *Video consultation with a counsellor/therapist*—84% found helpful). In contrast, although *Talking to a ChatBOT, text counselling* and *self‐help apps* had been used by more, they were perceived as less helpful—14%, 34% and 47%, respectively, found them helpful.

Some of the underlying reasons for young people's preferences were elaborated on in their free‐text comments. For example, the least popular type of support was Chatbots, with only 14% of users finding them helpful. Five negative comments showed strong sentiment, for example: ‘I hate computer bots’ (#443: NZE, 18–19 years, woman) and one participant specified the lack of connection when using one as a reason for his dislike:

Being online, speaking to AI just doesn't feel real, it's hard to gain connection.

(#739: NZE, 18–19 years, man).

In contrast, however, two comments indicated benefits for some to be found in the anonymity and lack of judgement of a Chatbot:

I found talking to a chatbot useful because I always have the lingering fear of being judged in the back of my head and I felt like it's a bot so it can't necessarily judge. I was able to customise the app to what I found most effective and helpful.

(#1102: NZE, 15–17 years, woman)

Chatbots would be better if they weren't so repetitive, although they provide a level of anonymity not found with actual therapists, which can sometimes be a good thing.

(#182: Māori, 20–21 years, other gender)

Online gaming that is designed as a form of mental health support (e.g., SPARX, a local example) rated in the middle of the pack in terms of popularity (67% finding it helpful) and has a strong appeal for some young people, with comments from both men and women in support of it:

Online gaming is certainly the most effective for me, subsequently becoming the most frequent form of digital support.

(#127: Māori, 15–17 years, man)

Games such as Sparx can be really helpful to redirect your attention when you're struggling.

(#202: European, 15–17 years, woman)

However, another comment indicated the limitations of games and a sense from young people that connection with real people in real life (IRL) is of greater importance (also indicated elsewhere in the findings):

Online games and media content provide some sense of hope such as relation of circumstances with fellow users but a lot of that is temporary support and finding real mutually beneficial and supportive relationships that way can be hard. (it's good that they are non‐committal and usually anonymous which is cool if you don't feel like reaching out to people you know …

(#245: European, 15–17 years, woman)

Websites were the most used digital medium, with 58% of users finding them helpful. Nonetheless, some respondents exhibited frustration with the poor design and lack of clarity of some websites or the resources:

The website ‘mental health.org.nz’ isn't easy to use. The ‘suicide prevention’ leads to only phone numbers and a 24 minute video no one has watched or wants to watch (it has less than 2000 views). I don't want to go through a labyrinth of pages and skim every page hoping to find something useful. Making the website more straight forward to use … would help.

(#541: Other European, 22–23 years, man)

More specific ‘straight to the point' sources for information. Having to read an entire article just to get the 1 paragraph of useful information can certainly stifle you from looking any further as it is time consuming.

(#757: Māori, 15–17 years, man)

In addition, some felt that the aim of some websites needed to be more clearly communicated:

Would prefer for services to explicitly state if they're there to listen/discuss your issues and feelings or if they're solely there to present all the resources available to you.

(#853: Māori, 24–25 years, man)

Some free‐text comments also addressed what features of digital mental health supports were felt to be important. One emphasised the need for them to be free or low cost, and of high quality:

it's more about not being able to find *good* digital support, instead of the more superficial types where it just tells you to drink more water or something.

(#1464: NZ European, 15–17 years, woman)

Another noted a need for a range of offerings to cater for different individuals:

also a wider range of free platforms so people can find what fits them best.

(#30: Asian, 15–17 years, woman)

One respondent felt that services in general were not well‐matched to the needs of young people:

Most of it doesn't meet the needs of young people. There's a large disconnect with what people need and with what people think we need.

(#535: NZ European, 18–19 years, man)

#### 3.2.3 Support People Would Like to Try

**Figure 3:**
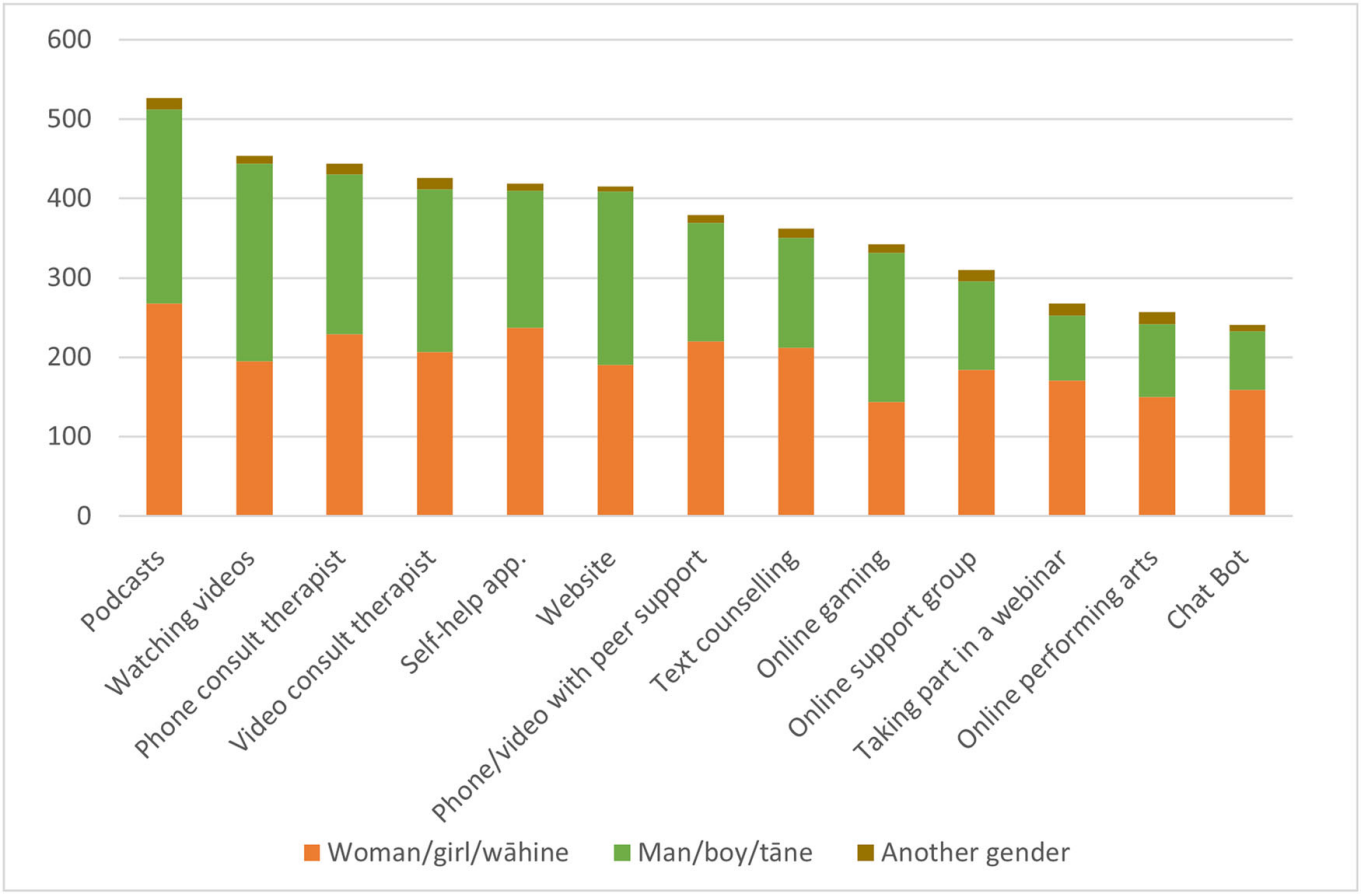
Digital support that people wanted to try (and had not tried before) by gender; participants could select as many as they liked.

Figure 3 shows the type of digital support that young people wanted to try (and had not tried before) by gender. *Listening to podcasts* was the most popular to try, followed by a *phone consultation with a counsellor or therapist. Talking to a ChatBOT* and *Online performing arts* were the least preferred. Respondents were also able to suggest in free text other options not covered in this list; these included talking to ‘online friends’ or ‘friends online’, ‘listening to music’, ‘messaging a friend’ and ‘VR (virtual reality) chat’.

Although overall there were more women than men who were interested in trying most types of digital support, more men than women were interested in watching videos (249 men cf 195 women), websites (219 men cf 190 women) and online gaming (188 men cf 144 women). This may reflect the general tendency for women to be more inclined to seek support and a possible appeal to men of mediums of support that require less personal input. It was difficult to note any patterns for those identifying as ‘another gender’ due to small numbers.

### 3.3 Methods of Letting Young People Know About Mental Health Supports

**Figure 4:**
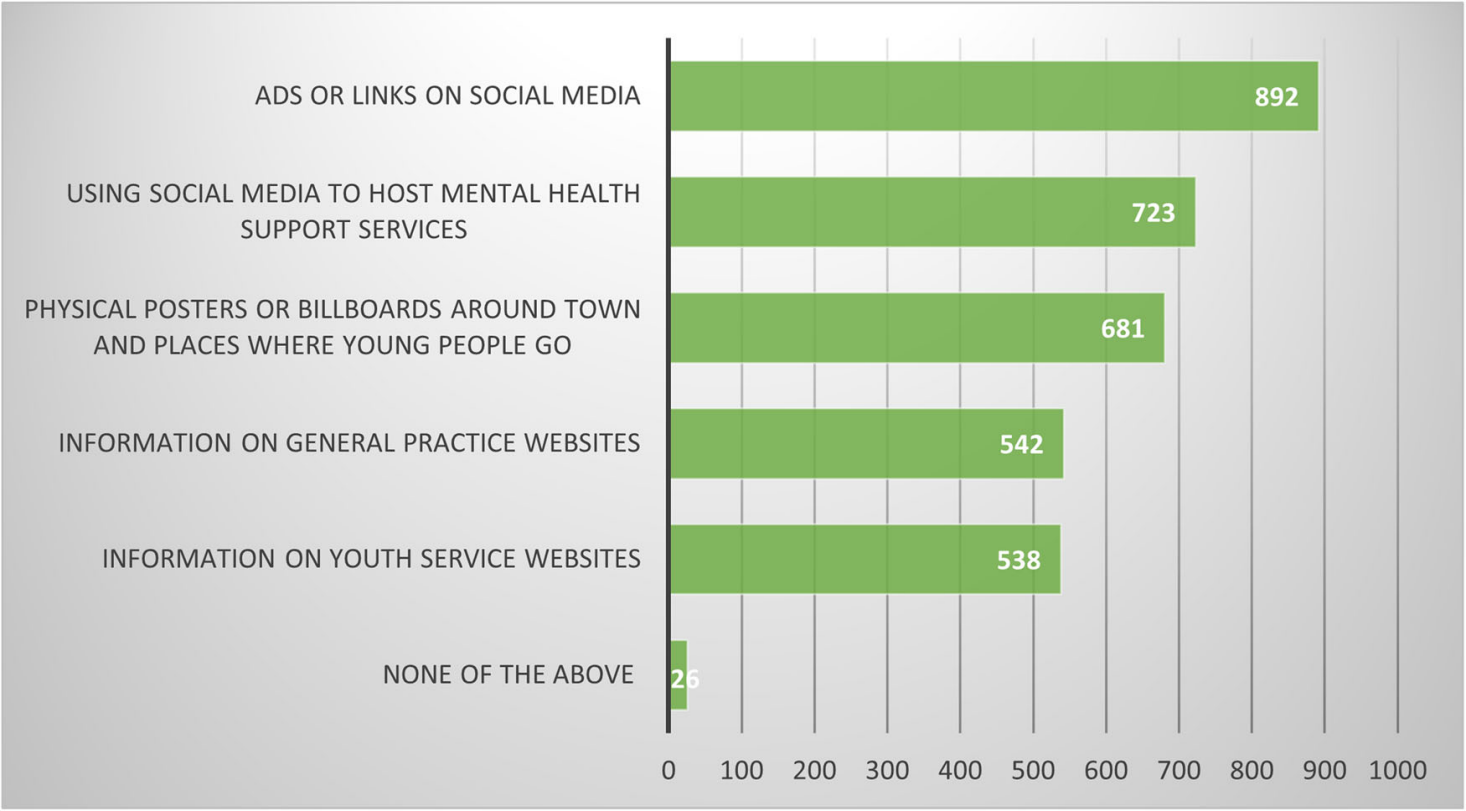
Preferred ways of letting young people know about mental health support; participants could select as many as they liked (*n* = 1045).

Respondents were asked, as an open free‐text question, how they would get the word out to other young people about what mental health support options are available (748 free‐text responses were provided). Following this, they were asked to select as many options as they liked from a list of five that they considered to be helpful. The results of the multiple‐choice question are shown in Figure 4.

The use of social media (including ‘Ads or links via social media’ and ‘using social media to host mental health support services’) was the most favoured means of letting people know about mental health support, both in the multiple choice and the preceding free‐text question responses. Provision of information on the websites of general practices or youth services was less preferred in the multi‐choice question and not mentioned in the free text.

#### 3.3.1 Digital Promotion

The following examples illustrate the appeal of relatable content presented on social media:

Hooks? Whatever those are called. Click bait? Like what buzzfeed does to draw people into clicking links. Idk [I don't know] relatable things associated to mental health issues that could make people think oh that's me! Haha relatable let's look at that!

(#558: Māori, 15–17 years, woman)

Social media may also be where some young people go when they are ‘not doing well' and may thus be a good place to find help at a time when it is needed:

In my opinion Instagram ads are really useful because idk [I don't know] I feel like when I'm not doing well I go on my phone and go through Instagram stories and basically the only ads I click on are through Instagram like this one was …

(#33: Māori, 15–17 years, woman)

Others noted that caution is necessary, with some indicating that social media should not be relied on to the exclusion of other modes of communication. There was also strong support for word of mouth and the importance of real people for communicating to young people about mental health supports (as for the support itself):

I think Facebook ads and etc are too easily skipped over for not being genuine.

(#1165: NZE, 24–25 years, man)

I honestly think word of mouth is still the best way. I'm much more likely to seek out an option when someone I trust recommends it (rather than an ad or similar). It feels weird to have such an intimate service recommended by an algorithm.

(#428: European, 22–23 years, man)

Free‐text comments indicated the value placed on trusted recommendation sources (sometimes in comparison to ads). Such sources included people they have one‐to‐one relationships with, most commonly friends but also family and GPs to a lesser extent:

An ad does not mean as much as a friend.

(#1122: NZE, 18–19 years, man)

voice of word from family.

(#1479: Pasifika, 15–17 years, man)

Trusted persons also included less intimate figures such as champions or influencers in the community or online.

finding big influencers/celebrities to promote mental health awareness and destigmatize MH and linking MH digital health support in their bios, making them like speakspersons [spokespersons].

(#309: Asian, 15–17 years, woman)

Celebrities or sports role models, as well as local community leaders, were also valued by some, sometimes by the same respondent:

Through leaders in the community. Older brothers, older sisters, people who have status in the community not just a public figure. If my older brother told me to access mental health support then I would do it. If Sonny Bill Williams [sport celebrity] told me to access the support I wouldn't jump at the idea straight away.

(#634: Māori, 24–25 years, man)

Communication from those with similar experiences, that is, with lived experience of mental distress and support, was also noted as important for their real‐life credibility. Some valued commentary from other young people:

I feel like I gravitate more towards young people speaking/giving their testimonials because even though it's been produced a LITTLE BIT probably like they've tried the service and it just gives it more credibility, especially when you can tell that they're being genuine and also not like speaking down to you which I see a lot in those videos

(#33: Māori, 15–17 years, woman)

Promotion through well‐known young New Zealanders. People that are the same age and ‘look like them’.

(#994: Māori, 26‐30 years, woman)

Others were not as concerned with the age of those sharing their experience:

Role models to highlight options—Men even when looking for help want to do it with dignity. If someone they admire/display qualities they admire talk about a shared experience then they are more likely to follow that path.

(#696: Pasifika, 24–25 years, man)

I found YouTube helpful. Just because I liked hearing of other people's real life experiences.

(#1501: Pasifika (Samoan/Tongan), 26–30 years, woman)

#### 3.3.2 Non‐Digital Promotion

The promotion of mental health supports in non‐digital spaces was also suggested by respondents. This included both written and oral communication encompassing posters/billboards and emails as well as brief talks within lectures or more sustained education on a par with physical education. A range of physical locations for messaging were mentioned, including workplaces, sports and recreation locations, youth events, pharmacies and youth health clinics. However, educational institutions were the most commonly mentioned:

The best thing at [tertiary education institute] was when the student advisor visited our class at the beginning of the year to tell us about counselling services and she said that 34 of 120 people in the previous cohort had accessed support through the counselling available there. It really helped to normalise that support is available and people do use it

(#148: NZE, 26–30 years, woman)

it always strikes me as bizarre that they would make us learn physical education where we'd learn just sports, and other health stuff like sexual health, but never anything about mental health, mental health disorders, how to prevent these, and what support is available—especially given how common these are.

(#1037: NZE, 22–23 years, man)

Several respondents suggested sustained, large‐scale campaigns to promote supports available for youth mental health issues, drawing a parallel with the recent campaigns promoting Covid‐19 vaccination, with one putting out a call for young men in particular to be targeted:

promote it in schools and ads on tv and youtube just like the government did for vaccines. It was sad to see how much effort they put in and initiatives they used for something like vaccines, but cannot do the same for poor mental health ….

(#1457: Māori, 26–30 years, woman)

… Potentially launch a product nationwide that's relatable to young men at risk with mental health … I feel as if our government spending should be on advertising options of how to get online help for young boys.

(#1104: Other European, 20–21 years, man)

#### 3.3.3 Constructing the Right Message

The need for high‐quality content and messaging with the right pitch, tone and wording was emphasised by a number of respondents, noting that messages need to be up to date, and to look ‘cool’, ‘catchy’, ‘click‐baity’, ‘eye‐catching’ and not ‘cheesy’ or ‘cringey’. The need to use simple straightforward language that also normalises seeking support and avoids the stigma was also noted.

Stop making the support sites so ‘soft and fluffy’ present facts and be more straightforward.

(#819: NZE, 20–25 years, man)

A catchy phrase that could type in to google search that doesn't feel so scary/overwhelming. Sometimes it can be hard to type ‘mental health help’ or ‘I'm depressed what do I do’.

(#994: Māori, 26–30 years, woman)

The use of co‐design methods was suggested by one person who identified as a Pasifika man:

Get them to be present in the design and roll out. Taking a collaborative approach to how it is made and then presented and promoted.

(#1454: Pasifika, 26–30 years, man)

The fit of services for specific youth sectors such as Māori/Pasifika and men was noted as often being poor:

To acknowledge that there is online platforms for me. I often discount stuff that I can't see myself using or see myself represented in fully and not in a tokenist way either. Such as sticking a brown guy on something doesn't work.

(#1454: Pasifika, 26–30 years, man)

Most mental health support is not geared towards modern men issues and often overlooked.

(#1020: NZE, 20–21 years, man)

Several respondents pointed out, that the best method of communication depends on a range of factors:

I think it depends on the person, situation and your mood because there will be times you really want to speak to someone or in other cases accessing it online …

(#135: Pasifika, 24–25 years, woman)

Or as one respondent simply put it:

there is no one way.

(#708: Māori, 24–25 years, man).

## 4 Discussion

In this survey of 1471 young people aged between 15 and 30 years, digital mental health tools were thought to be most useful for self‐help and for locating other supports. Some of the perceived disadvantages of using digital support included concerns over trust and privacy, a preference for in‐person support and the need for high levels of motivation to continue. The most common form of digital support already used was browsing a website, with podcasts identified as the support that people would most like to try (and had not tried). Views on different types of support were highly varied, with no one size fits all.

Social media was heavily favoured as a way of finding out about the types of support available, but not to the exclusion of word of mouth from friends/whanau, hearing from other young people who had already used supports or from respected figures such as community or sports leaders.

The free‐text comments reflected many of the known benefits and drawbacks of digital support noted in the introduction [1]. Other studies have similarly noted the complementary role that digital support can play with mental health as opposed to providing the only means of support [29], with web‐based self‐help, mobile self‐help and blended therapy favoured the most [30].

This survey also supports previous research findings that customising digital tools so that users can see themselves represented is important for minority or Indigenous groups [6]. Poor usability is the primary reason for discontinuation of mental health app use [6] and some of our respondents also noted this for websites: ‘I don't want to go through a labyrinth of pages … hoping to find something useful’.

Factors to consider for digital mental health interventions for young people include tailoring the intervention to the target audience [31]. Our respondents wanted to see something relatable that would them make think ‘oh that's me’. Co‐design with youth is an important way to achieve this [32] as well as to create appropriate promotion of such services, as mentioned by several respondents (e.g., celebrities, trusted community figures, siblings) and supported by our youth co‐researchers. Respondents mentioned having the ability to customise apps as a useful feature. Providing an interpersonal connection and feeling of belonging [33] can also be achieved with mediums such as online gaming, with the additional layer of anonymity in these domains also seen as useful by some for avoiding stigma.

A similar online survey undertaken in Ireland (*n* = 393) following lockdown found that respondents used social media (51.4%) and mental health apps (32.6%), with fewer making use of formal online resources such as professional counselling services (13.2%). In contrast, mental health apps were seldom mentioned by our respondents, which may be due to a wider variety of apps being available in the Northern hemisphere [15].

The desirability of a variety of methods for publicising support found in our survey was in line with previous studies, including recommendations from peers and those with prior knowledge of services [15] and including both digital and physical methods of promotion [32].

The fact that more women than men reported using digital mental health support most likely reflects the tendency for women to seek mental health support in general. Some of the most frequently used supports were those that are easy to provide and access, but these were not necessarily rated as the most helpful ones. This suggests that it is worth investing in more helpful types of support that may be more challenging and labour‐intensive to provide, such as online performing arts and phone or video counselling.

Overall, the theme of trustworthiness was evident, in the sense that young people are keen to hear from real people they trust, whether this is ‘in real life’ or via digital means, and whether they are personal contacts, influential figures such as celebrities or influencers and/or people who are relatable (either through having had similar experiences or looking like them). Digital supports also need to be transparently trustworthy and reliable, and security concerns need to be clearly and effectively dealt with. To better support young men and people of diverse ethnic backgrounds, digital supports and their promotion need to be framed carefully to avoid being ‘soft and fluffy’ and to balance the need to represent a diverse community without being seen as tokenistic. This requires going beyond imagery to making sure that the content is truly authentic and relevant to all.

### 4.1 Strengths and Limitations

Our study sample was large, demographically diverse and broadly reflective of the population of young people in New Zealand. We heard both from young people who had and those who had not used digital support for mental health, and thus obtained views of both ‘experienced’ and ‘potential’ users. The employment of a group of young people as co‐researchers to advise on survey design, advertising methods and wording may have strengthened the survey's appeal and helped to obtain a good response.

The methodology for recruiting a diverse mix of participants in terms of gender and ethnicity, using targeted advertising, meant that we recruited a high number of respondents identifying as men who are often under‐represented in such studies. Our recruitment method (use of social media advertising) was both a strength and a limitation of the study. This method allowed us to link with a good number of demographically diverse young people who would have been difficult to reach via other recruitment methods, but also meant that we may have excluded those with limited or no access to the internet or a device. Our findings might therefore be biased towards views held by people facing fewer social and economic challenges. However, a number of those who did respond noted challenges in their access to digital support.

Nearly 40% of people who clicked on the advertisement initiated a survey, though there was some dropoff in responses towards the end, resulting in missing demographic data. We sought to include Māori (Indigenous people of New Zealand) and Pasifika young people by targeting these groups specifically in our ads during the second burst of the campaign. This resulted in the proportion of respondents in these two groups being similar to general population proportion rates. Survey uptake among young people in rural areas was low, with respondents heavily weighted towards those living in large urban areas. We did not include questions in the survey on whether participants had any disability that may have prevented them easily accessing digital support.

## 5 Conclusions

This study confirms previous research findings that digital support should not replace real‐life mental well‐being support. Although young people in our study recognised that digital support has a place in mental health care, they expressed a strong sentiment in favour of in‐person support and the need for trustworthiness, high‐quality, tailored supports and messaging (whether in person or digital). Some found digital support beneficial, but it was evident that for many, its value was more limited, for example, as a non‐threatening starting point, for locating other supports and for integrating with real life support. The most frequently used digital supports (e.g., browsing websites, watching videos) were not necessarily rated as the most helpful. Less frequently used supports (e.g., phone or video consults with a counsellor and online performance arts) were nevertheless highly rated, and podcasts may also be a new area of support to explore for this population group.

Overall, the findings suggest that digital mental health supports for youth are never ‘one size fits all’ and it should not be assumed that digital support or digital modes of publicising will be universally effective for this generation of ‘digital natives.’

## Author Contributions

**Susan M. Garrett:** data curation, formal analysis, visualisation, writing–original draft, writing–review and editing, validation, investigation, methodology, conceptualisation. **Jo Hilder:** data curation, formal analysis, writing–original draft, writing–review and editing, investigation, methodology, visualisation. **Rachel Tester:** conceptualisation, data curation, formal analysis, visualisation, writing–review and editing, project administration, investigation. **Abby Dunlop:** data curation, formal analysis, writing–review and editing, investigation. **Tracey Gardiner:** data curation, formal analysis, writing–review and editing, project administration, investigation. **Tony Dowell:** conceptualisation, writing–review and editing, investigation. **Soraya Kamau Brady:** methodology, writing–review and editing, investigation, validation, conceptualisation. **Nicole Gilbert:** methodology, investigation, writing–review and editing, validation, conceptualisation. **Maggie Shippam:** writing–review and editing, methodology, investigation, validation, conceptualisation. **Shay Tanirau:** methodology, investigation, writing–review and editing, validation, conceptualisation. **Neo Kenny:** methodology, investigation, writing–review and editing, validation, conceptualisation. **Caitlin McBride:** methodology, investigation, writing–review and editing, validation, conceptualisation. **Joana Wilson:** methodology, investigation, writing–review and editing, validation, conceptualisation. **Ellie Rukuwai:** conceptualisation, methodology, investigation, writing–review and editing, validation. **Niusha Aryan:** conceptualisation, methodology, investigation, writing–review and editing, validation. **Maria Stubbe:** conceptualisation, funding acquisition, data curation, formal analysis, writing–review and editing, supervision, investigation, methodology, writing–original draft.

## Consent

Informed consent was obtained from each participant as per the ethical approval granted by the University of Otago Human Ethics Committee (Health), Ref: H21/103.

## Conflicts of Interest

The authors declare no conflicts of interest.

## Supporting information

Supporting information.

## Data Availability

The data supporting this study are unable to be submitted to a public repository as participants in the study did not consent to this.
