# Supplementary material for: Young People Talk About Digital Support for Mental Health: An Online Survey of 15–30‐Year Olds in New Zealand
Source: Health Expect. 2024 Aug 26;27(4):e70001. doi: 10.1111/hex.70001 (PMC11347751; doi:10.1111/hex.70001)
Supplement: Supplementary file 1 — Supporting information. [file HEX-27-e70001-s001.pdf]

Intro Block

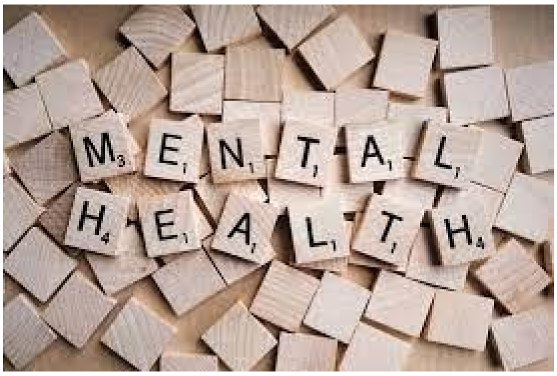

Survey: How would you like to find information about mental health support for you or a friend?

About this survey

What's it about? We want to find out what young people think about various places or ways someone can find information about how to get support for mental health.

How long is it? About 5-7 minutes.

Agreement to take part (Consent). Starting the survey will tell us that you agree to take part. You can stop it at any time and can go back in to finish the survey if you get interrupted but you will need to bookmark or save the link.

How will this information be used? It will help us to improve access to information for young people needing mental health support. Your responses will be completely anonymous.

What do I get out of taking part? You can go into the draw to win one of six $50 prizes when you've finished the survey.

Who is doing this research? Researchers from the University of Otago in Wellington including a community-based group of 15-25 year olds.

How is it funded? By a Lottery Health Research Grant

Ethical approval. This study has been approved by the University of Otago Human Ethics Committee Health (ref: H21/103, Dec 2020). Email gary.witte@otago.ac.nz if you have any concerns.

Click here to download a copy of our Information Sheet: Information Sheet

Main Block

Please tell us a little about yourself. We want to make sure we hear from a range of young people.

How old are you? *Please note this survey is only for 15 - 30 year olds.*

- ○ 15 - 17 years
- ○ 18 - 19 years
- ○ 20 - 21 years
- ○ 22 - 23 years
- ○ 24 - 25 years
- ○ 26 - 30 years

What is your gender?

- ○ Woman/girl/wāhine
- ○ Man/boy/tāne
- ○ [ ] Another gender, please say if you would like:
- ○ Choose not to answer

Which ethnic group do you belong to? *Select all that apply to you.*

- ☐ Māori
- ☐ Samoan
- ☐ Cook Island Māori
- ☐ Tongan
- ☐ Niuean
- ☐ Chinese
- ☐ Indian
- ☐ New Zealand European
- ☐ Other such as Dutch, Japanese, Tokelauan, please state: [ ]
- ☐ Choose not to answer

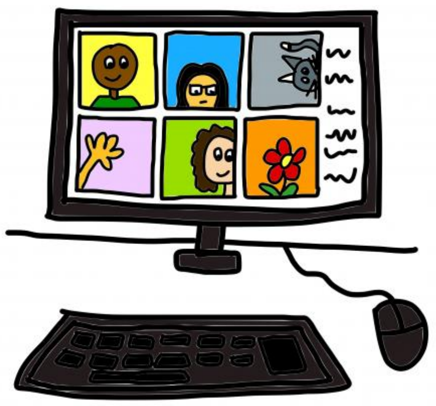

Have you used digital support for mental health before?

By "digital support" we mean any kind of mental health support where you are not with a real person in the same space. This can include talking to a real person but over a device (phone, video, text, email, messaging, community forum) as well as anything online such as websites, apps, games or chat-bots.

- ○ Yes
- ○ No

Please tick below which forms of digital support you have used and whether it was helpful OR if you haven't used it, whether you might try it.

	Have used before	Found it helpful	Have not used but would be something I would try
Website (e.g. reading information, looking at images and other non-interactive activity)	☐	☐	☐
Watching videos on platforms such as YouTube, Instagram	☐	☐	☐
Listening to podcasts	☐	☐	☐
Self-help app (e.g. mood tracker, sleep diary)	☐	☐	☐

	Have used before	Found it helpful	Have not used but would be something I would try
Talking to a chat bot	☐	☐	☐
Phone consult with counsellor or therapist	☐	☐	☐
Video consult with counsellor or therapist	☐	☐	☐
Phone/video consult with peer support worker	☐	☐	☐
Text counselling	☐	☐	☐
Online support group or other community forums	☐	☐	☐
Taking part in a webinar	☐	☐	☐
Online gaming	☐	☐	☐
Online performing arts (e.g. skits, artwork, songs/music, stories)	☐	☐	☐
Anything else you've used, please say:	☐	☐	☐

Any comments you would like to add about what you have tried, what you found helpful or what you would like to try?

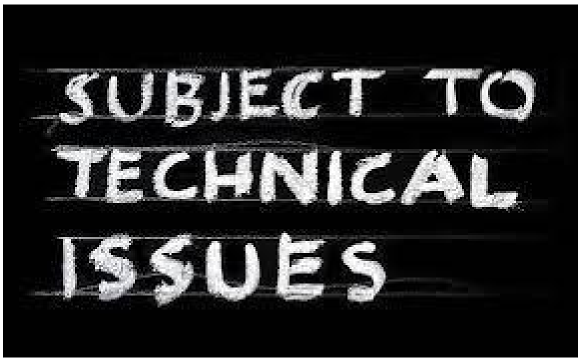

Have you ever had problems accessing digital support? Select all that apply to you.

- ☐ I've never had problems accessing digital support
- ☐ Poor internet connection
- ☐ Lack of privacy at home
- ☐ Concerns with data security
- ☐ Cost
- ☐ Not enough data
- ☐ No suitable device
- ☐ Prefer in-person support
- ☐ I don't know where to find digital support
- ☐ Other problem, please say:

Is there anything that would have improved your experience of using digital support? Select all that apply.

- ☐ Having an existing relationship with the support person
- ☐ Being able to see the person you're talking to
- ☐ Having free or better access to a device

- ☐ Support person available sooner/when you need them
- ☐ Being able to talk to or message a real person as well as doing stuff by myself
- ☐ None of the above
- ☐ Other, please say:

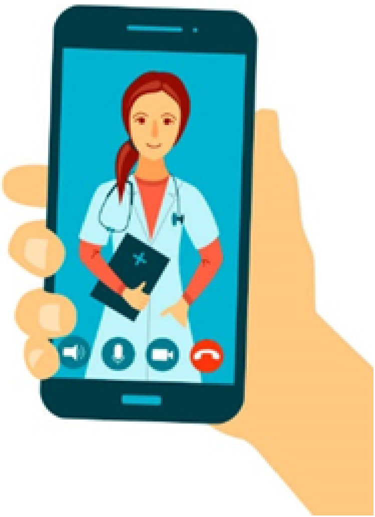

Below are some types of digital support that are available. If you needed to use digital support for mental health, please indicate which ones you would consider trying? Select all that apply.

- ☐ Website (e.g. reading information, looking at images and other non-interactive activity)
- ☐ Watching videos on platforms such as YouTube, Instagram
- ☐ Listening to podcasts
- ☐ Self-help app (e.g. mood tracker, sleep diary)
- ☐ Talking to a chat bot
- ☐ Phone consult with counsellor or therapist
- ☐ Video consult with counsellor or therapist
- ☐ Phone/video consult with peer support worker

☐ Text counselling

☐ Online support group or other community forum

☐ Taking part in a webinar

☐ Online gaming

☐ Online performing arts (e.g. skits, artwork, songs/music, stories)

☐ None of the above

☐ Anything else you think you would like to try, please say:

What do you think digital forms of mental health support might be useful for? Select all that apply.

☐ Finding the best place to access support

☐ Self-help

☐ An extra support when also using in-person therapy

☐ Useful for therapy/peer support when you find it difficult to do in-person

☐ Help me figure out what kind of support would suit me best

☐ To get support right away

☐ None of the above

☐ Other, please say:

How would you get the word out to other young people about what options are available for mental health support?

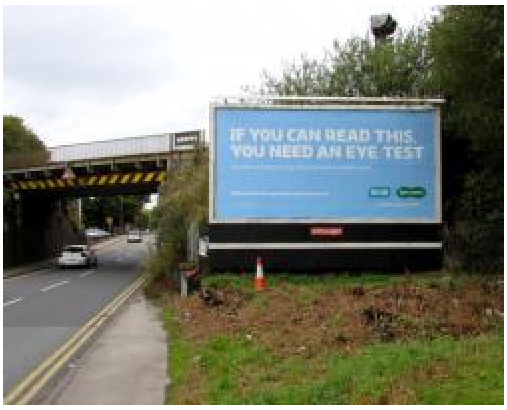

Do you think any of the following would be helpful to get the word out to other young people about what's available for mental health support? Select all that apply.

- [ ] Physical posters or billboards around town and places where young people go
- [ ] Ads or links on social media
- [ ] Using social media to host mental health support services
- [ ] Information on youth service websites
- [ ] Information on general practice websites
- [ ] None of the above

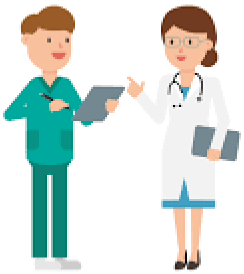

If you found yourself or a friend needing to access support for mental health, where do you think you would go to get information?

Select all that apply.

- ☐ GP or practice nurse
- ☐ Friends
- ☐ Family
- ☐ Counsellor (school, college, university, work)
- ☐ Online search
- ☐ None of the above
- ☐ Other, please say:

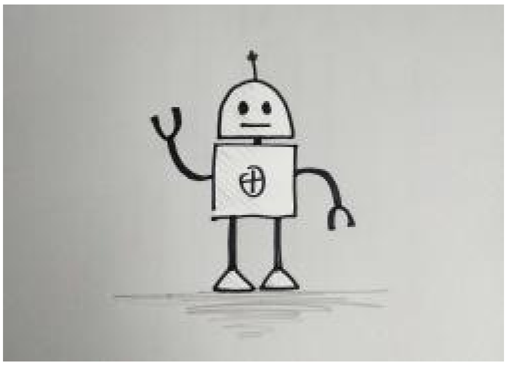

If you didn't want to ask someone in person about where to find mental health support, where would you prefer to go to find this out? Rank the following in order of preference or give your own suggestion.

	1	2	3	4	5	6	7
A website	○	○	○	○	○	○	○
An app	○	○	○	○	○	○	○
An online chat bot that you could ask	○	○	○	○	○	○	○

	1	2	3	4	5	6	7
A dedicated social media site	○	○	○	○	○	○	○
A phone number to ring to ask	○	○	○	○	○	○	○
An online quiz to help you work out the best option	○	○	○	○	○	○	○
Other, please say:	○	○	○	○	○	○	○

What would help you to find digital modes of mental health support? Give us your ideas.

Final Questions

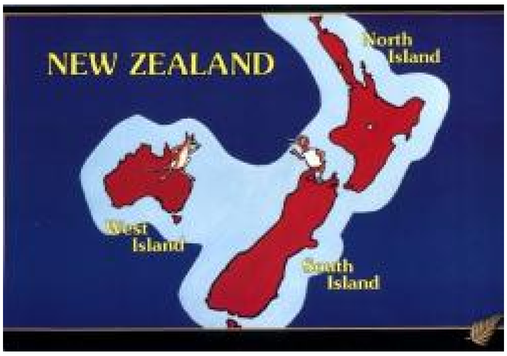

Please tell us a bit more about yourself so we can describe the group of people taking part in this survey.

Which region do you live in?

- ○ Northland/ Te Tai Tokerau
- ○ Auckland/ Tāmaki-makau-rau
- ○ Waikato
- ○ Bay of Plenty/ Te Moana-a-Toi
- ○ Gisborne/ Te Tai Rāwhiti
- ○ Hawkes Bay/ Te Matau-a-Māui
- ○ Taranaki
- ○ Manawatu-Whanganui
- ○ Wellington/ Te Whanganui-a-Tara
- ○ Tasman/ Te Tai-o-Aorere
- ○ Nelson/ Whakatū
- ○ Marlborough/ Te Tauihu-o-te-waka
- ○ West Coast/ Te Tai Poutini
- ○ Canterbury/ Waitaha
- ○ Otago/ Ōtākou
- ○ Southland/ Murihiku

Which of these best describes you? *Select all that apply to you.*

- ☐ At school or studying at polytech or university
- ☐ Training/apprenticeship
- ☐ Working part time
- ☐ Working full time
- ☐ Looking for work
- ☐ Caring for children or another person
- ☐ Not working or studying
- ☐ Unable to work or study
- ☐ Receiving a benefit
- ☐ Other, please say; ____________

☐ Choose not to answer

Block 2

Do you live:

- ◯ In a major urban city (e.g. Auckland, Wellington, Christchurch, Hamilton, Dunedin)
- ◯ In a regional city (e.g. Nelson, Whangarei, New Plymouth, Timaru)
- ◯ In a small town (e.g. Levin, Cambridge, Blenheim, Oamaru)
- ◯ In a very small town/rural/remote (e.g. Wairoa, Pahiatua, Lumsden)

Powered by Qualtrics
